# Few-Layer MoS_2_ Nanodomains Decorating TiO_2_ Nanoparticles: A Case Study for the Photodegradation of Carbamazepine

**DOI:** 10.3390/nano8040207

**Published:** 2018-03-29

**Authors:** Sara Cravanzola, Marco Sarro, Federico Cesano, Paola Calza, Domenica Scarano

**Affiliations:** Department of Chemistry, NIS (Nanostructured Interfaces and Surfaces) Inter-Departmental Centre and INSTM Centro di Riferimento, University of Torino, Via P. Giuria, 7, 10125 Torino, Italy; sara.cravanzola@unito.it (S.C.); marco.sarro@unito.it (M.S.); domenica.scarano@unito.it (D.S.)

**Keywords:** TiO_2_, MoS_2_, hybrid materials, photodegradation, carbamazepine, transmission electron microscopy, Raman spectroscopy, UV-Vis spectroscopy, FTIR spectroscopy, photocatalytic activity

## Abstract

S-doped TiO_2_ and hybrid MoS_2_/TiO_2_ systems have been synthesized, via the sulfidation with H_2_S of the bare TiO_2_ and of MoO*_x_* supported on TiO_2_ systems, with the aim of enhancing the photocatalytic properties of TiO_2_ for the degradation of carbamazepine, an anticonvulsant drug, whose residues and metabolites are usually inefficiently removed in wastewater treatment plants. The focus of this study is to find a relationship between the morphology/structure/surface properties and photoactivity. The full characterization of samples reveals the strong effects of the H_2_S action on the properties of TiO_2_, with the formation of defects at the surface, as shown by transmission electron microscopy (TEM) and infrared spectroscopy (IR), while also the optical properties are strongly affected by the sulfidation treatment, with changes in the electronic states of TiO_2_. Meanwhile, the formation of small and thin few-layer MoS_2_ domains, decorating the TiO_2_ surface, is evidenced by both high-resolution transmission electron microscopy (HRTEM) and UV-Vis/Raman spectroscopies, while Fourier-transform infrared (FTIR) spectra give insights into the nature of Ti and Mo surface sites. The most interesting findings of our research are the enhanced photoactivity of the MoS_2_/TiO_2_ hybrid photocatalyst toward the carbamazepine mineralization. Surprisingly, the formation of hazardous compounds (i.e., acridine derivatives), usually obtained from carbamazepine, is precluded when treated with MoS_2_/TiO_2_ systems.

## 1. Introduction

Nowadays, titanium dioxide (TiO_2_) is a well-known material, whose characteristics, i.e., non-toxicity, excellent chemical stability and low cost [1,2] make its applications in the photocatalysis field highly widespread, also in industries. For these reasons, the interest of scientific research is strongly focused on studying and enhancing the properties of such a material, with the aim to improve its performances.

As a matter of fact, a huge variety of works reported in the scientific literature deals with TiO_2_-based materials for photocatalytic applications [3]. In particular, the modification of titania and the tunneling of its characteristics are still considered challenging objectives, because TiO_2_, despite its amazing properties, only absorbs a small part of the solar spectrum in the UV range [4]. Therefore, the engineering of its band gap can help in shifting its absorption to the visible light region, in such a way as to improve the potentialities of its applications. An additional drawback of titania is the fast charge recombination, which involves the electron/hole pairs [5], even though it is not an easy issue to clarify the relationship between the photocatalytic activity of a metal oxide and its morphological and electronic properties [6].

However, in order to overcome these difficulties and enhance TiO_2_ performances, many methods have been proposed.

Beyond the development of TiO_2_-based materials with a variety of morphologies [4,7,8,9,10], doping is undoubtedly an effective way to modify the absorption band gap of a semiconductor, tailoring its properties according to the required final performances [3]. Among the suitable dopants, able to introduce the desired band gap, both metals and non-metals (nitrogen [10,11,12], iodine [13,14], fluorine [15,16] and carbon [17,18,19]) have been widely studied [20,21]. In particular, among non-metals [22], sulfur is considered a promising candidate for the tuning the band gap in TiO_2_ [23,24]. Different from other heteroatomic counterparts, sulfur is for a fact isoelectronic to oxygen and can replace it without altering the electroneutrality of the solid [3]. It has been proven that the treatment of a TiO_2_ surface under H_2_S atmosphere can induce a modification of the titania electronic structure, causing a red shift of its absorption edge [25]. Some articles [26,27,28] suggest that MoS_2_/TiO_2_ composites are good photocatalysts, thanks to the efficient charge-carrier separation [29]. Along with this line of thinking, H_2_S, S, CS_2_, etc., have been used to create hybrid heterojunctions. In fact, the 2D layered MoS_2_, a well-known transition metal dichalcogenide whose band gap increases, decreasing its layer number, provides effective electron transfer [30].

For this reason, MoS_2_/TiO_2_ systems are widely studied in photocatalysis, with applications in different fields, from the production of lithium-ion batteries [31,32,33], to H_2_ generation [3,34,35], hydrodesulfurization (HDS) [28] and degradation of organic dyes [4,36,37].

As far as the field of photocatalysis is concerned, much attention has been given to the use of TiO_2_ for the degradation of pharmaceutical residues and their metabolites, as nowadays, the production, synthesis and use of drugs is under continuous development. These pollutants mainly come from pharmaceutical industries and medical excretory products, and their presence in both surface and drinking water is a growing environmental concern. Moreover, they are often inefficiently removed in wastewater treatment plants. For these reasons, the studies concerning water treatments for the degradation of pharmaceuticals, by taking advantage of the photocatalytic properties of TiO_2_, is very rich [38,39,40].

Carbamazepine (CBZ) is an anticonvulsant drug used for the treatment of epilepsy, bipolar disorder and trigeminal neuralgia [41], and its occurrence in surface waters has frequently been reported [42,43,44,45].

To the best of our knowledge, there is a gap in the literature concerning the use of TiO_2_ based on hybrid interfaces with MoS_2_ for CBZ photodegradation applications.

Following this line, in this work, the performances in the photocatalytic degradation of carbamazepine for sulfided TiO_2_ and MoS_2_/TiO_2_, both obtained by a thermal treatment under H_2_S atmosphere, are compared with the benchmark TiO_2_ P25. The transformation products formed along with CBZ degradation and the total organic carbon (TOC) are also evaluated and reveal the absence of hazardous compounds (i.e., acridine derivatives) that usually are obtained when carbamazepine is degraded by traditional TiO_2_ materials.

The different catalysts are characterized by transmission electron microscopy (TEM), Raman, UV-visible and Fourier-transform infrared (FTIR) spectroscopies.

## 2. Materials and Methods

### 2.1. Materials

Carbamazepine (CBZ) analytical standard (purity ≥99%), HPLC-grade acetonitrile (purity ≥99.9%), formic acid (purity ≥85%) and phosphoric acid (purity ≥85%) were obtained from Sigma-Aldrich (Milan, Italy) and used as received. All solutions and eluents were prepared with ultrapure water Millipore Milli-Q™ (Millipore Co., Bedford, MA, USA) (TOC < 2 ppb, conductivity ≥18 MΩ cm).

#### 2.1.1. Synthesis of MoO_x_/TiO_2_ Samples

A water solution of ammonium heptamolybdate (AHM, Merck, Milan, Italy), 0.066 g in 1 mL of water solution, was added drop by drop to 2 g TiO_2_ (P25, Evonik, Pandino, Italy), by following a wet impregnation method. In order to remove the solvent, the impregnated powder was then dried in air overnight. The final concentration of molybdenum was about 3 wt %. In order to decompose the AHM and to remove ammonia and water, the obtained MoO_x_ samples followed a preliminary air thermal treatment into a muffle furnace at 673 K for 12 h.

#### 2.1.2. Samples Activation and Sulfidation

MoO*_x_*/TiO_2_ (P25) samples, in the form of pellets, were activated under dynamic vacuum at 673 K for 30 min and then oxidized in an oxygen (40 Torr) atmosphere at the same temperature for 30 min, twice. By keeping the temperature at 673 K, the oxidized samples were sulfided in a H_2_S (30 Torr) atmosphere for 1 h, then outgassed. The samples were successively further sulfided, following the same procedure, in such a way so as to obtain a MoS_2_/TiO_2_ sample. Pure TiO_2_ was also sulfided following the same procedure.

### 2.2. Methods

Transmission electron microscopy (TEM) images were acquired with a JEOL 3010-UHR instrument (JEOL Ltd., Tokyo, Japan) operating at 300 kV, equipped with a 2 k × 2 k pixel Gatan US1000 CCD camera.

Raman spectra were recorded by using a Renishaw Raman InVia Reflex spectrophotometer (Renishaw plc, Wotton-Under-Edge, UK) equipped with an Ar^+^ laser emitting at 442 nm, using both static and rotating configurations.

X-ray diffraction (XRD) patterns were collected by means of a diffractometer (PANalytical PW3050/60 X’Pert PRO MPD, PANalytical, Almelo, The Netherlands) with a Ni-filtered Cu anode, working with a reflectance Bragg–Brentano geometry, by using the spinner mode.

UV-Vis spectra on samples were collected by using a Varian DRUV Cary 5000 spectrometer (Varian Inc., Palo Alto, CA, USA), equipped with a diffuse reflectance sphere. Due to their strong optical absorption, the samples were diluted in BaSO_4_ powder. FTIR spectra of CO (70 Torr) adsorbed at 77 K on TiO_2_ and MoS_2_/TiO_2_ at decreasing coverages were obtained in an IR cell designed for liquid nitrogen flowing and were recorded by means of a Bruker IFS-28 spectrometer (Bruker Optics, Karlsruhe, Germany), equipped with a mercury cadmium telluride (MCT) cryogenic detector, with a resolution of 4 cm^−1^ (64 interferograms were averaged for each spectrum). The spectra were acquired in the 4000–400-cm^−1^ interval, where the fundamental vibration modes are observed. N_2_ adsorption-desorption experiments have been carried out at 77 K (Micromeritics ASAP 2020 instrument, Micromeritics, Norcross, GA, USA) to determine the Brunauer–Emmett–Teller (BET) surface area. The surface area of the samples was determined after outgassing at RT, overnight.

#### 2.2.1. Carbamazepine Photodegradation Tests

##### Irradiation Procedures

The photocatalytic performance of the catalysts was assessed through the photocatalytic degradation of carbamazepine (CBZ). Experiments have been carried out in air-saturated Pyrex glass cells, filled with 5 mL of CBZ (10 mg/L) and the catalyst (500 mg/L). Irradiations were performed using a Philips TLK/05 lamp (Monza, Italy) of 40 Watts with maximum emission at 360 nm. Samples were subjected to different irradiation times (times ranging from 5 min to 3 h) and then filtered through 0.45-µm Millipore disks to remove catalyst particles.

##### Analytical Procedures

The degradation of CBZ was monitored by using a Merck-Hitachi liquid chromatographer (Merck-Hitachi, Tokyo, Japan) equipped with a Rheodyne injector L-6200 and L-6200A pumps for high-pressure gradients, an L-4200 UV-Vis detector (the detection wavelength was set at 210 nm) and a column LiChrocart RP-C18 (Merck, 12.5 cm × 0.4 cm). Isocratic elution (1 mL/min flow rate) was carried out with 45% of phosphate buffer 1 × 10^−2^ M at pH 2.8 and 55% acetonitrile; in these conditions, the retention time for CBZ was 3.56 min.

Transformation products were identified by using high performance liquid chromatography-high resolution mass spectrometry (HPLC/HRMS). The chromatographic separations were achieved with a Phenomenex Luna 150 mm × 2.1 mm, 3 µm, using an Ultimate 3000 HPLC instrument (Thermo Scientific, Milan, Italy). The injection volume was set at 20 µL and the flow rate at 0.2 mL/min. A gradient mobile phase composition was adopted: 5/95–95/5 for 45 min in acetonitrile/formic acid 0.05% in water. A LTQ Orbitrap mass spectrometer (Thermo Scientific, Bremen, Germany) equipped with an atmospheric pressure interface and an ESI ion source was used. All samples were analyzed in ESI positive mode. The LC column effluent was delivered into the ion source using nitrogen as both the sheath and auxiliary gas. The tuning parameters adopted for the ESI source were: capillary voltage 31.00 V, tube lens 135 V. The source voltage was set to 4.5 kV. The heated capillary temperature was maintained at 270 °C. Analyses were run using full mass (50–1000 *m*/*z*) range with a resolution of 30,000 in FTMS mode. Xcalibur (Thermo Scientific, Bremen, Germany) software was used both for acquisition and for elaboration.

Total organic carbon (TOC) was measured on filtered suspensions using a Shimadzu TOC-5000 analyzer (catalytic oxidation on Pt at 680 °C). The calibration was performed using standards of potassium phthalate.

## 3. Results and Discussion

### 3.1. Morphology and Structure of Samples

**Figure 1:**
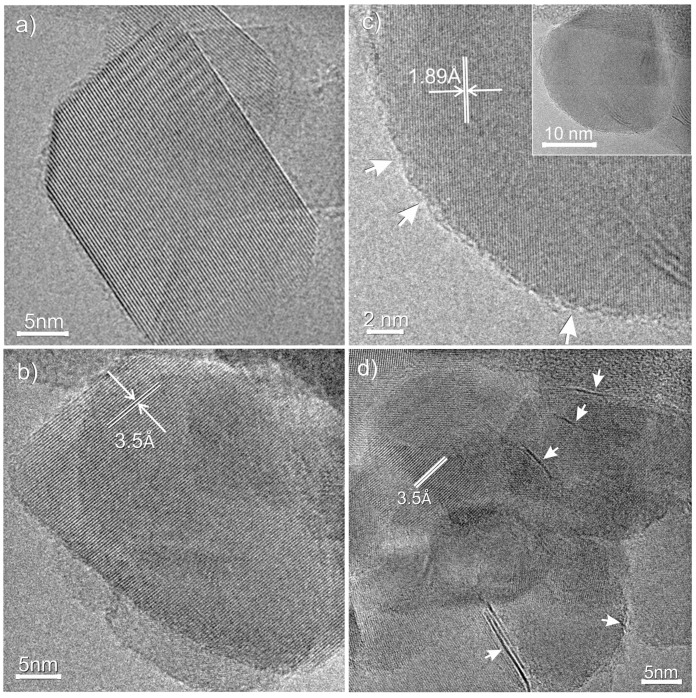
HRTEM images of (**a**) TiO_2_ P25, (**b**) sulfided-TiO_2_ and (**c**, **d**) MoS_2_/TiO_2_ (Mo 3 wt %). The arrows in (**c**) indicate local erosions on TiO_2_ particles.

High-resolution TEM images of TiO_2_ P25, sulfided TiO_2_ and MoS_2_/TiO_2_ are shown in Figure 1.

From Figure 1a, it can be observed that the native TiO_2_ particles show a well-defined structure and shape, with extended faces, highly regular terminations, sharp corners and edges (see also [46]). From the comparison of Figure 1b (sulfided TiO_2_) and Figure 1c, d (MoS_2_/TiO_2_) with that of pure TiO_2_ P25 (Figure 1a), it is clear that the action of H_2_S at 400 °C gives rise to the formation of a TiO_2_ defective surface, where the presence of rounded terminations, due to the formation of local defective regions, can be highlighted. In particular, the anatase particle, exposing (101) faces (Figure 1b), shows local erosions at the atomic level (i.e., indented borders, corners and sensitively smoothed edges).

Similar TiO_2_ morphologies are also observed on the MoS_2_/TiO_2_ sample, together with the presence of curved and differently-oriented few-layer-thick MoS_2_ nanosheets, from 1 to 3 layers in thickness, decorating the TiO_2_ particles and showing (200) lattice fringes, thus indicating the interaction between the MoS_2_ nanosheets and the support (inset in Figure 1c,d). A more detailed discussion on the morphology of the few-layer MoS_2_ nanosheets (i.e., curvature, surface detects, decoration of nanoparticles, etc.), as a result of the interaction of MoS_2_ with the support, is beyond the scope of this work and can be found elsewhere, but we shall return later to the MoS_2_/support interaction (see the FTIR section).

**Figure 2:**
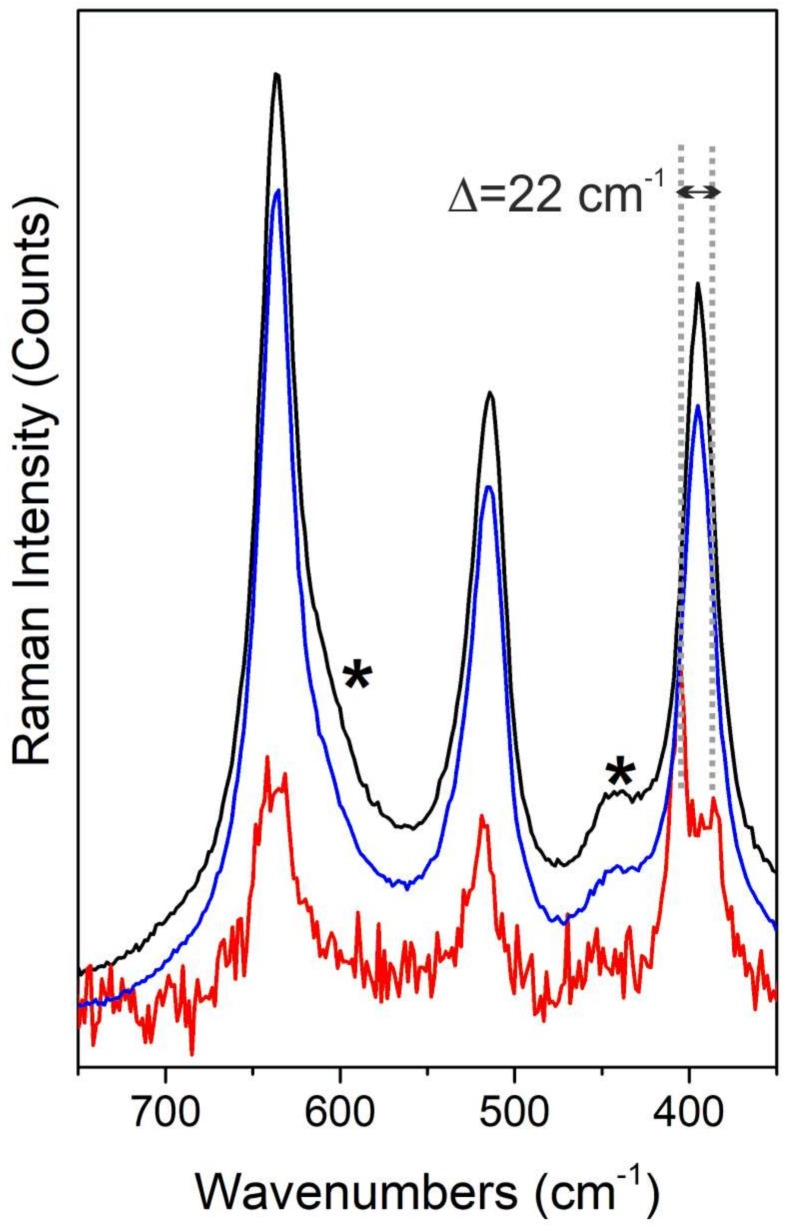
Raman spectra, acquired with a 442-nm laser line, of: TiO_2_ used as a reference material (black line), sulfided TiO_2_ (blue line) and MoS_2_/TiO_2_ (red line) samples. Raman fingerprints of rutile are marked by asterisks.

In Figure 2, the Raman spectra, recorded with a 442-nm laser line, of sulfided TiO_2_ (blue line) and MoS_2_/TiO_2_ (red line) samples are compared with pure TiO_2_ P25 (black line), used as a reference material.

Concerning the spectrum of pure TiO_2_ in the 750–350-cm^−1^ range (Figure 2, black curve), the bands at 144 cm^−1^, 396 cm^−1^, 514 cm^−1^ and 636 cm^−1^ are assigned, respectively, to the typical E_g_, B_1g_, A_1g_ and E_g_ Raman active modes, respectively, of the anatase phase, as well described in literature, while the shoulder at 608 cm^−1^ and the very small peak at 444 cm^−1^ (highlighted by asterisks) are ascribed to the A_1g_ and E_g_ modes of the rutile phase [8,25,27].

These typical TiO_2_ fingerprints are also clearly present in the sulfided sample (blue line), while further new modes are not observed after the sulfidation step.

On the other hand, on the MoS_2_/TiO_2_ sample (red line), besides the Raman modes of TiO_2_ previously described, it can be observed that the feature centered at 396 cm^−1^, assigned to TiO_2_ anatase phase, is split into two components at 405 cm^−1^ and at 384 cm^−1^, which can be ascribed to the A_1g_ and E^1^_2g_ first-order Raman active modes of MoS_2_. As well reported in the literature, the difference of the frequency values between A_1g_ and E^1^_2g_ modes is indicative of the thickness of the MoS_2_ particles. In particular, a stacking number of 2 ± 1 layers, calculated from Δ = 21 ± 1 cm^−1^ [26], confirms the results obtained by TEM images. Furthermore, the presence of very thin MoS_2_ nanosheets (mainly mono-, bi- and few-stacked layers) has been confirmed by XRD analyses (Figure S1, Supplementary Materials). Going into detail, the XRD pattern of MoS_2_/TiO_2_ (red pattern) shows also two minor diffraction peaks (marked with asterisks in the inset) at 2θ = 31.2° and 2θ = 59.0° assigned to the (100) and (110) diffraction planes of the hexagonal MoS_2._ The presence of these two features together with the absence in the low angles region (2θ ≈ 14°) of the diffraction planes along the *c*-axis (i.e., 002) that are associated with the stacking of the MoS_2_ layers means that very thin MoS_2_ slabs, too thin to be detected by XRD, are formed.

### 3.2. Optical and Surface Properties of Samples

**Figure 3:**
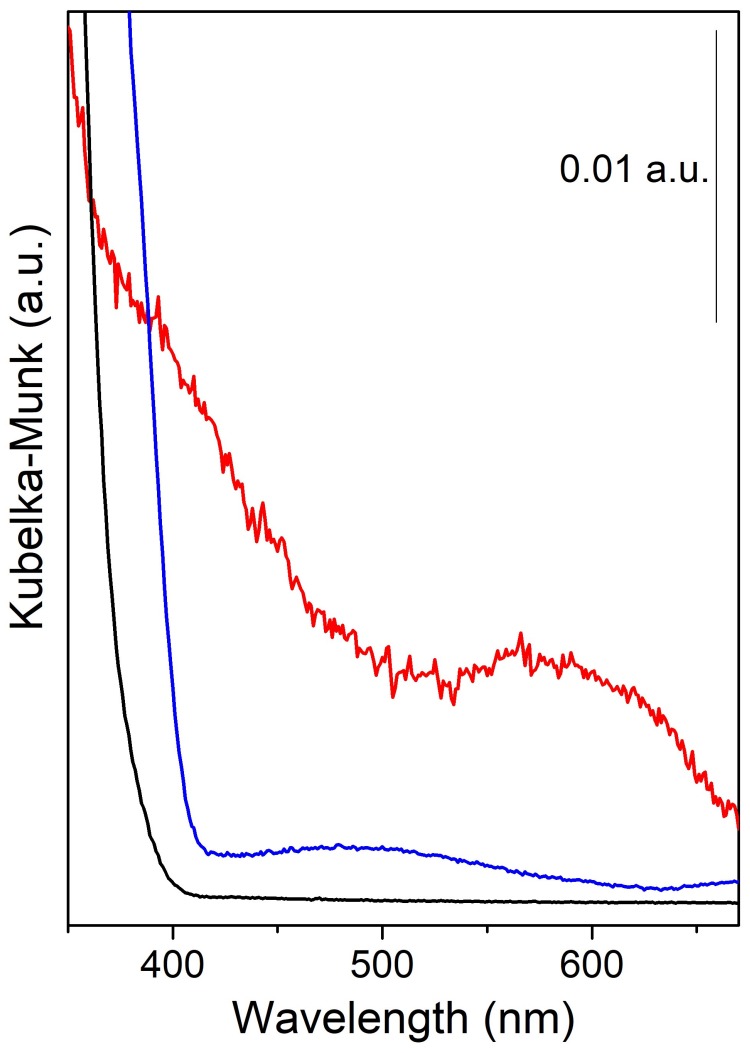
UV-Vis spectra of MoS_2_/TiO_2_ (red curve), sulfided-TiO_2_ (blue curve) and pure TiO_2_ used as a reference (black curve).

The optical properties of the samples were investigated by comparing the UV-Vis spectra of MoS_2_/TiO_2_, sulfided TiO_2_ and pure TiO_2_, as shown in Figure 3 (red, blue and black curves, respectively). From the comparison of the spectra of TiO_2_ before and after the sulfidation treatment (black and blue curves, respectively), a clear band gap shift of the sulfided TiO_2_ sample, well shown by a Tauc plot in [25], together with an additional broad absorption in the 390–600-nm range are detectable. As well described in the literature [47] and in our previous work [25], these phenomena can be ascribed to a change in TiO_2_ electronic structure. In fact, the mixing of 3p atomic orbitals of S species, due to a S → O exchange at the surface of TiO_2_ during the sulfidation step, as well discussed in the following paragraphs, leads to the formation, above the valence band, of new electronic states [3,25,47].

Besides, when the MoS_2_/TiO_2_ spectrum is considered, the presence of MoS_2_ gives rise to some new features in the visible-light region, specifically in the 400–450 nm and 500–690 nm ranges.

Going into detail, the modes at about 680 nm and at 600 nm have been ascribed to the typical MoS_2_ A and B excitonic transitions, respectively, whose separation in energy can be related to the spin-orbit splitting at the top of the valence band at the K point of the Brillouin zone [30]. Furthermore, the wide band in the 400–450 interval, can be explained as an envelope due to other excitonic transitions of MoS_2_, named as C and D, in this case strongly affected by the absorption edge of TiO_2_ [30].

**Figure 4:**
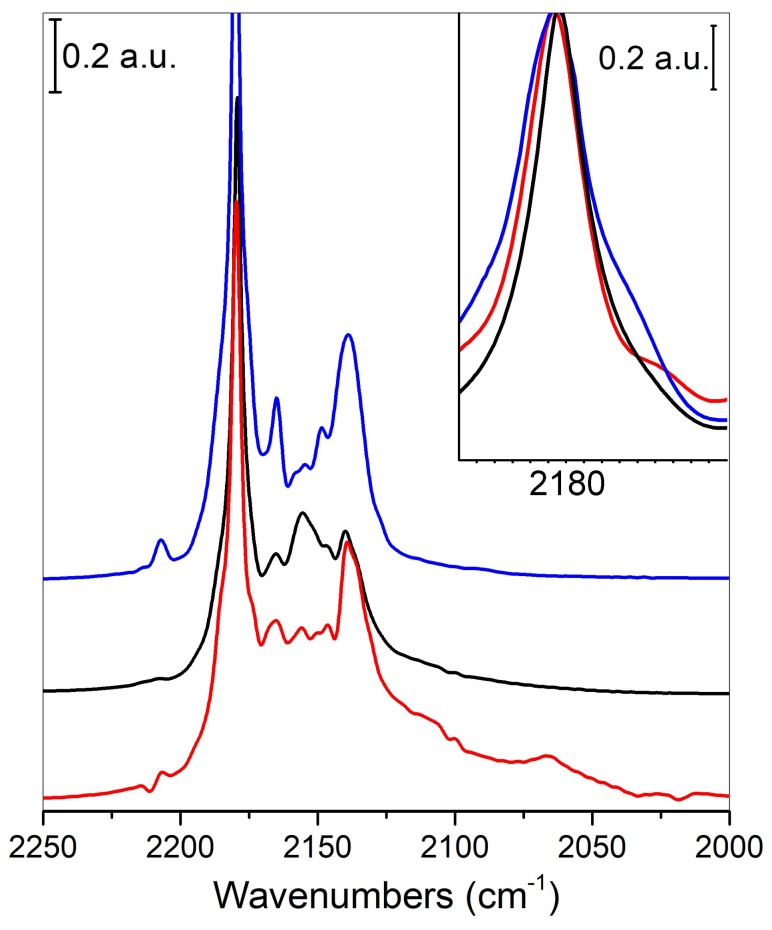
IR spectra at the maximum coverage of CO, adsorbed at the surface at 77 K of MoS_2_/TiO_2_ (red curve), TiO_2_ P25 (black curve) and sulfided-TiO_2_ (blue curve). In the insert, an exploded view of the main feature centered at 2178 cm^−1^.

FTIR spectra of 70 Torr CO adsorbed at 77 K on TiO_2_, sulfided TiO_2_ and MoS_2_/TiO_2_ are compared in Figure 4 (black, blue and red curves, respectively), aiming to investigate the effects of the sulfidation step and of the presence of MoS_2_ on the surface properties of TiO_2_.

For a clear understanding of the IR features of the synthesized sample, a brief analysis of the pure TiO_2_ spectrum (black curve) has to be done. First of all, the main features are due to the typical adsorptions of CO on the different Ti sites of the TiO_2_ surface. Specifically, the intense main peak centered at 2178 cm^−1^ can be explained with the building up of parallel CO oscillators interacting with five-fold coordinated Ti_5c_^4+^ sites located on flat (101) surfaces [48], while the one at 2155 cm^−1^ is explained with hydrogen interactions of CO with residual OH groups, even present after the thermal treatment [25,27,48]. It is noteworthy that, even if not reported in Figure 4, for the sake of simplicity, the previously discussed 2178-cm^−1^ band shifts to higher frequencies, when CO pressure is decreased, due to the changes of the lateral interactions between adjacent CO molecules on the surface of TiO_2_. This phenomenon is indicative of a highly extended and regular face [25].

Moving to the sharp band at 2140 cm^−1^, it is assigned to CO in a “liquid-like” state, caused by liquid nitrogen temperature and physically adsorbed as a multilayer surface [25,27], while the one at 2146 cm^−1^ is due to CO adsorbed on facelets of the rutile phase.

The feature at 2166 cm^−1^ is due to CO interacting with Ti Lewis centers on flat (001) faces, where Ti centers along Ti-O rows are strongly bonded to two O anions [49], causing at these sites a more screened electronic potential and therefore a reduced acidity.

Finally, the weak band at 2208 cm^−1^ is assigned to CO adsorbed on Ti Lewis acidic defective sites, such as steps, corners and edges, with higher coordinative unsaturations [48].

Along with TiO_2_ after H_2_S dosage (blue curve), it is worth noticing that all the observed features are indicative of a more disordered system. In particular, the wider full width at half maximum (FWHM) of the 2179 cm^−1^ band (as well shown in the inset of Figure 4) can be explained with the presence of S species that interrupt the regularity of the TiO_2_ (101) extended faces. H_2_S dosage also affects the 2166 cm^−1^ band, previously described, showing now an increased intensity due to a higher acidity, which has been explained with the O → S exchange reaction during the sulfidation treatment [25]. Moreover, the band at 2208 cm^−1^ is now higher in intensity, plausibly due to the presence of defective sites, such as large S atoms that, replacing the smaller O anions, favor the formation of corners, steps and edges [25].

In point of fact, a dissociative adsorption of H_2_S molecules takes place on the TiO_2_ surfaces via two main competitive routes, to produce H_2_O and oxygen vacancies or H_2_. According to some authors, in the first case, S may move into the O vacancy, giving rise to the S-doped TiO_2_ surface, whereas in the latter case, S-adatoms are formed [50,51].

Moreover, high-resolution X-ray photoelectron spectroscopy (XPS) spectra showed the presence of Si–Ti–O bonds, although the exact form of the S-doped structure (i.e., S-substitution or S-adatom) until now is not well understood. Conversely, on the basis of density functional theory (DFT) calculations [50,51] and even confirmed by IR data [52], it results that H_2_S adsorbed on TiO_2_ can be converted into S and H_2_O at a temperature above 473 K. Since in our case the samples are sulfided at 673 K, we can state that the O → S exchange is occurring, giving rise to S-substituted O_2c_ vacancies. Hence, it is expected that under the adopted synthesis parameters (i.e., 673 K, under vacuum), the MoS_2_ slabs lose sulfur, giving rise to vacancies formed at the most exposed sites, where sulfur anions show the lowest coordination, even if sulfur vacancies also on a flat surface are not excluded (vide infra)*.*

Moving to the interaction of CO with the MoS_2_/TiO_2_ surface (red curve), the general decrease in intensity can be associated with the presence of new Mo*^x^*^+^ species, masking the Ti^4+^ sites, which are no longer available for interactions with CO. The formation of new bands in the 2130–2000-cm^−1^ interval can confirm the presence of the aforementioned Mo*^x^*^+^ species (*x* < 4) [27]. Notice that both the 2109-cm^−1^ and the 2066-cm^−1^ bands are assigned to CO interacting with defective sites on edges and corners of MoS_2_ slabs. In particular, the band at 2109 cm^−1^ has been assigned to Mo*^x^*^+^ located on edges, including Mo oxysulfide species (MoO*_x_*S*_y_* phase), while the one at 2066 cm^−1^ is due to reduced Mo*^x^*^+^ species associated with surface sulfur vacancies on very exposed sites [26].

It is worth noticing that the band at 2208 cm^−1^, due to the presence of defective sites, although still present, is now considerably decreasing its intensity, thus restoring a situation similar to the pure TiO_2_. It can be hypothesized that the sulfur sites responsible for the increased disorder, and then for the increased intensity of the 2208 cm^−1^ band in the sulfided TiO_2_ sample, are now involved in the formation of MoS_2_ domains with Mo cations. Therefore, the sulfur sites are now masked by Mo*^x^*^+^ species, making them no more available for the interaction with CO. In the same way, the 2166-cm^−1^ band, already assigned to CO interacting with TiO_2_ (100) faces, is also decreasing in intensity, if compared to the same feature on sulfided TiO_2_, thus being now more similar to the TiO_2_ one. As explained before, this could be due to the involvement of S anions in the formation of MoS_2_ domains, thus restoring the initial acidic TiO_2_ conditions.

Coming back to the exact form of the S-doped structure (i.e., S-substitution or S-adatom), the role played by the support can be highlighted. As a matter of fact, from previous FTIR investigations of CO molecules interacting with different supports: MoS_2_/SiO_2_, γ-Al_2_O_3_ and MgO, it turns out that the sulfidation process has an influence on the support matrix. In fact, MgO incorporates sulfur in the structure; γ-Al_2_O_3_ shows a surface reactivity; while SiO_2_ does not show reactivity at all [26]. Some more incorporation of S^2−^ ions on the surface of the support, in turn, influences the MoS_2_/support interaction, which means that the interaction between MoS_2_ and the support, “acting as a chemical ligand”, increases by moving from SiO_2_, to γ-Al_2_O_3_, to MgO [26].

As for the MoS_2_/TiO_2_ interaction, we can find only a surface reactivity, because no formation of metal sulfide has been observed (as shown for MgO with the formation of crystalline MgS).

This remark is also in agreement with the moderate curvature observed for the few-layer MoS_2_ nanosheets, following the profile of the support particles (insert in Figure 1c,d). A more detailed discussion of this topic is beyond the scope of this work and can be found elsewhere [26].

### 3.3. Photocatalytic Activity

**Figure 5:**
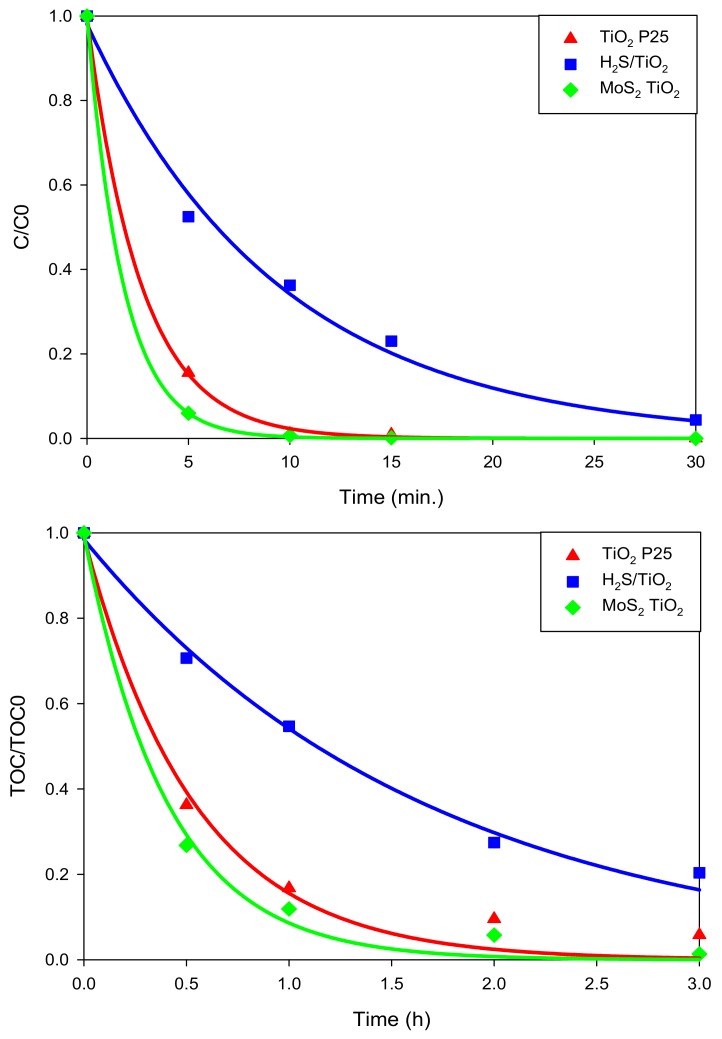
Carbamazepine (CBZ) degradation profiles (expressed as the ration between CBZ concentration after irradiation and CBZ at Time 0) (**top**) and TOC curves abatement (**bottom**, expressed as the ration between TOC after irradiation and TOC at Time 0).

The photocatalytic performance of the synthetized materials was tested on the abatement of carbamazepine (CBZ). Preliminarily, adsorption in the dark and direct photolysis for CBZ were investigated, and they were negligible in the considered time window (3 h) (Figure 5).

The disappearance profiles for CBZ over time are shown in top panel of Figure 5 and followed the order: MoS_2_/TiO_2_ > TiO_2_ P25 > sulfided TiO_2_. These results evidenced that starting from TiO_2_ P25, the photoactivity decreased during the treatment with H_2_S (the pseudo-first order kinetic constants decreased from 0.382 min^−1^ to 0.096 min^−1^), while the addition of MoS_2_ led to an enhancement of the photocatalytic activity (0.470 min^−1^).

The same trend was also maintained when analyzing the TOC disappearance profiles (Figure 5, bottom). In the presence of MoS_2_/TiO_2_, the complete mineralization was achieved within 3 h of irradiation; at that time, 20% and 5% of TOC are still present with the sulfided TiO_2_ and TiO_2_ P25 samples, respectively.

#### Carbamazepine Transformation Products’ Investigation

**Figure 6:**
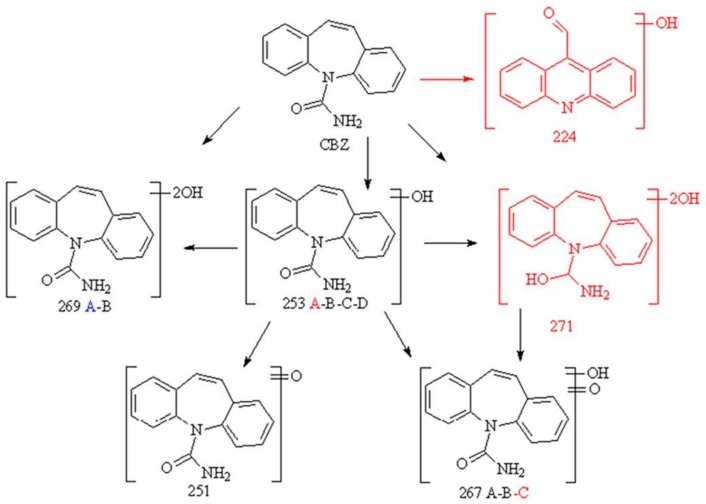
TPs identified during the degradation of CBZ. In black, TPs formed with all materials; in red, TPs characteristic of the TiO_2_ P25 degradation pathway; in blue, detected only with MoS_2_/TiO_2_ and/or sulfided TiO_2_ samples.

The transformation products (TPs) formed along with CBZ degradation are collected in Table 1, and their structures are shown in Figure 6.

Some peculiar differences arose for the three catalysts. Even if hydroxylation (and further oxidation of hydroxyl group(s)) seemed to be the favorite transformation pathway and all materials led to the formation of several (poly)hydroxylated and (poly)hydroxylated/oxidized derivatives (253.0977, 251.0891, 269.0935, 267.0786 and 271.1081 *m*/*z*), the largest number of hydroxylated products is formed with TiO_2_ P25. These transformation products were already detected and characterized during photocatalytic treatment of CBZ [53] and in river waters [42]. Analyzing sulfided TiO_2_, the TPs are formed to a lesser extent and with minor isobaric species compared to MoS_2_/TiO_2_ and TiO_2_ P25.

Two TPs were formed in the presence of TiO_2_ P25 only at 224.0710 and 223.1039 *m*/*z*, while their formation was precluded with MoS_2_/TiO_2_ and sulfided TiO_2_ samples. The one at 224 is a hydroxylated acridine-9-carboxaldehyde [54] whose formation involved the 7C ring CBZ contraction with the formation of a 6C ring.

It is well known that TiO_2_ P25, thanks to its excellent performance, is widely used as a reference material in photocatalysis. It is noteworthy that the decreased CBZ photodegradation ability of the sulfided TiO_2_ could be affected in part by the interruption of the regularity and order of the strongly active TiO_2_ faces, as previously demonstrated by FTIR investigation. The increment of disorder caused by the formation of defects, such as edges and corners, after the treatment with H_2_S, does not play a positive role in improving the photocatalytic properties of TiO_2_, rather causing a worsening of the performances of the material. Conversely, the improvement of the photodegradation properties has been observed for the MoS_2_/TiO_2_ sample. Among the reasons for such higher photoactivity, the masking effect of the defective S sites by Mo*^x^*^+^ species can be assumed firstly. Secondly, the intimate phase junction between MoS_2_ and TiO_2_ nanoparticles goes here beyond the simple physical contact (i.e., reduction of the electron-hole recombination, electrons/holes mobility across heterojunctions) [3]. The role played by the second semiconductor, composed of very small and thin MoS_2_ slabs dispersed on the surface of TiO_2_ nanoparticles, could be here viewed as that of a photosensitizer (i.e., charge injection of excited MoS_2_ and improved efficiency of electron transfer from the sensitized MoS_2_ to TiO_2_ nanoparticles) [55,56]. Lastly, it can be hypothesized that the proven presence of Mo*^x^*^+^ (*x* < 4) centers, on the slabs with their semiconductor nature, play a key role in the enhancement of the results, notwithstanding the specific surface area (Table 2). In this regard, the decreased surface area of sulfided TiO_2_ and MoS_2_/TiO_2_, compared to TiO_2_ P25, can find an explanation in the moderate sintering effect caused by the annealing conditions obtained during the treatments with H_2_S at high temperature. Even so, the variations of the surface area do not seem to follow and affect the trend obtained for the abatement of CBZ.

It is worth noticing that these systems have been also tested for the photodegradation of methylene blue (MB) in water solution, under a solar light simulating irradiation. The results (see Supplementary Materials, Figure S2) show the same trend obtained for the abatement of CBZ, making the MoS_2_/TiO_2_ sample a versatile material for applications in the field of photodegradation of organic pollutants in water.

## 4. Conclusions

In this work, S-doped TiO_2_ and MoS_2_/TiO_2_ hybrid systems have been synthesized and fully characterized, with the aim to enhance the well-known photocatalytic properties of P25 TiO_2_ and tested for the photodegradation of carbamazepine. The investigations performed by HRTEM clearly showed the morphology of TiO_2_ particles, on which the action of H_2_S gave rise to surface defects. However, the formation of MoS_2_ slabs, 1–3 layers in thickness, decorating the planes of TiO_2_, has been highlighted. FTIR results gave a further demonstration of the severe action of H_2_S, with changes in the relative intensity of all the peaks of the S-TiO_2_ sample, if compared to pure TiO_2_, and the formation of new features assigned to the formation of defective sites. However, the disorder degree seems to decrease for the hybrid MoS_2_/TiO_2_ sample, thus hypothesizing that the sulfur defective sites have a role in the formation of MoS_2_ with Mo cations.

Finally, the strong effects of H_2_S treatment have been also attested by UV-Vis spectra, which show how the sulfiding agent can affect the TiO_2_ electronic structure, with a shift of its band gap and, then, the appearance of the typical MoS_2_ excitonic modes.

As for the photocatalytic properties of the samples for the degradation of CBZ, the photoactivity decreased for the sulfided TiO_2_, while an enhancement has been shown by MoS_2_/TiO_2_, when compared to the benchmark TiO_2_. The same trend was observed when analyzing the TOC disappearance profiles.

Results highlighted that, even if all materials lead to the formation of transformation products, the largest number of hydroxylated products is formed with pure TiO_2_. As opposed to pure TiO_2_, only hybrid MoS_2_/TiO_2_ and sulfided TiO_2_ precluded the formation of acridine derivatives, representing an important improvement for the treatment of CBZ.

## Figures and Tables

**Table 1:** CBZ and transformation products (TPs) identified during the photocatalytic experiments with the three materials (area detected after 15 min of irradiation).

[M + H]^+^	Name	*t*_R_ (min)	TiO_2_ P25 (Area)	Sulfided-TiO_2_ (Area)	MoS_2_/TiO_2_ (Area)
237.1025	CBZ	21.33	1.07 × 10^9^	1.17 × 10^9^	8.71 × 10^8^
253.0977	253-A	14.77	8.39 × 10^6^	n.d.	n.d.
253.0977	253-B	17.30	5.37 × 10^7^	2.89 × 10^7^	5.51 × 10^7^
253.0977	253-C	18.34	5.41 × 10^7^	3.01 × 10^7^	5.22 × 10^7^
253.0977	253-D	19.08	6.98 × 10^6^	2.19 × 10^6^	3.60 × 10^6^
251.0891	251	16.95	6.26 × 10^7^	3.16 × 10^7^	8.90 × 10^7^
269.0935	269-A	15.57	n.d.	n.d.	3.51 × 10^6^
269.0935	269-B	16.79	5.15 × 10^6^	n.d.	5.02 × 10^6^
267.0786	267-A	15.25	1.28 × 10^6^	6.26 × 10^5^	2.29 × 10^6^
267.0786	267-B	16.49	1.36 × 10^6^	1.98 × 10^6^	3.37 × 10^6^
267.0786	267-C	18.51	6.24 × 10^5^	n.d.	n.d.
271.1081	271	14.77	6.62 × 10^6^	n.d.	n.d.
224.0710	224	22.40	8.19 × 10^6^	n.d.	n.d.

**Table 2:** Surface area properties.

Sample	*S*_BET_ (m^2^/g)
TiO_2_	55
sulfided TiO_2_	38
MoS_2_/TiO_2_	37

## Supplementary Materials

The following are available online at http://www.mdpi.com/2079-4991/8/4/207/s1, Figure S1: XRD patterns of samples; Figure S2: Evolution of UV-Vis spectra of MB in water exposed under visible light.
