# Supplementary material for: Few-Layer MoS2 Nanodomains Decorating TiO2 Nanoparticles: A Case Study for the Photodegradation of Carbamazepine
Source: Nanomaterials (Basel). 2018 Mar 29;8(4):207. doi: 10.3390/nano8040207 (PMC5923537; doi:10.3390/nano8040207)
Supplement: Supplementary file 1 [file nanomaterials-08-00207-s001.pdf]

Few-Layer MoS_2 Nanodomains Decorating TiO_2 Nanoparticles: A Case Study for the Photodegradation of Carbamazepine

Sara Cravanzola[1], Marco Sarro[1], Federico Cesano[1]*, Paola Calza[1]* and Domenica Scarano [1]

[1] Department of Chemistry, NIS (Nanostructured Interfaces and Surfaces) Inter-Dipartimental Centre and INSTM Centro di Riferimento, University of Torino, Via P. Giuria, 7, 10125 Torino (Italy).

* Correspondence: federico.cesano@unito.it; paola.calza@unito.it; Tel.: +39 011 670 7834/5268

Supplementary Materials

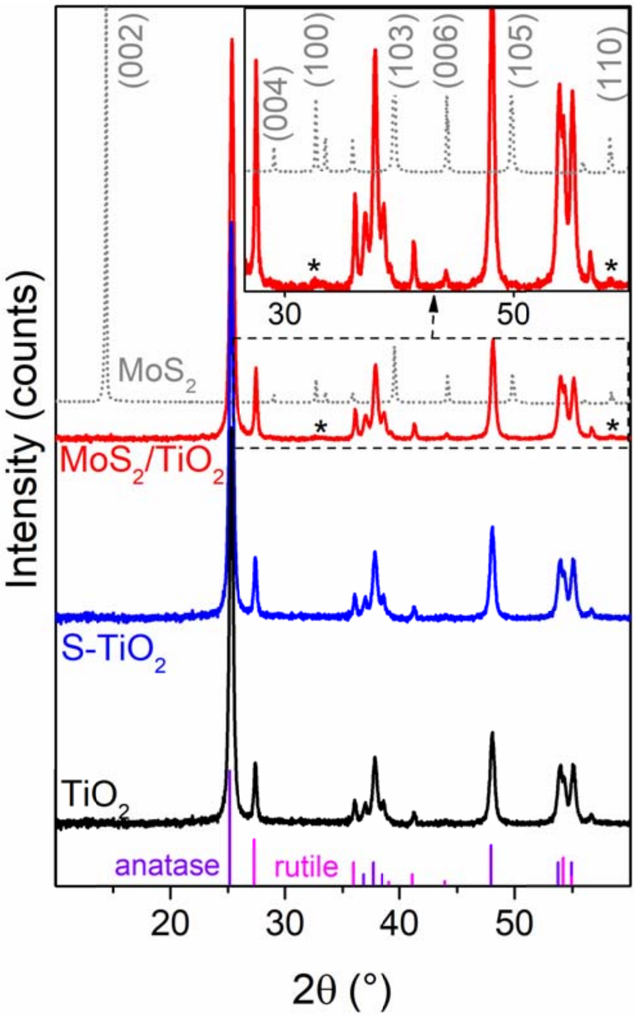

Figure S1. XRD patterns of TiO_2 (black curve), H_2S/TiO_2 (blue curve), MoS_2/TiO_2 (red curve) and of microcrystalline hexagonal MoS_2 powder used as a reference (dotted gray curve). Anatase (PDF card n.#21-1272) and rutile (PDF card n. #21-1276) peak positions (violet and magenta lines, respectively) are shown for comparison. In the inset, MoS_2/TiO_2 vs. microcrystalline MoS_2 patterns is shown in the $25<2\theta$ (°) <47 interval. Asterisks in the figure refer to (100) diffraction planes of hexagonal MoS_2.

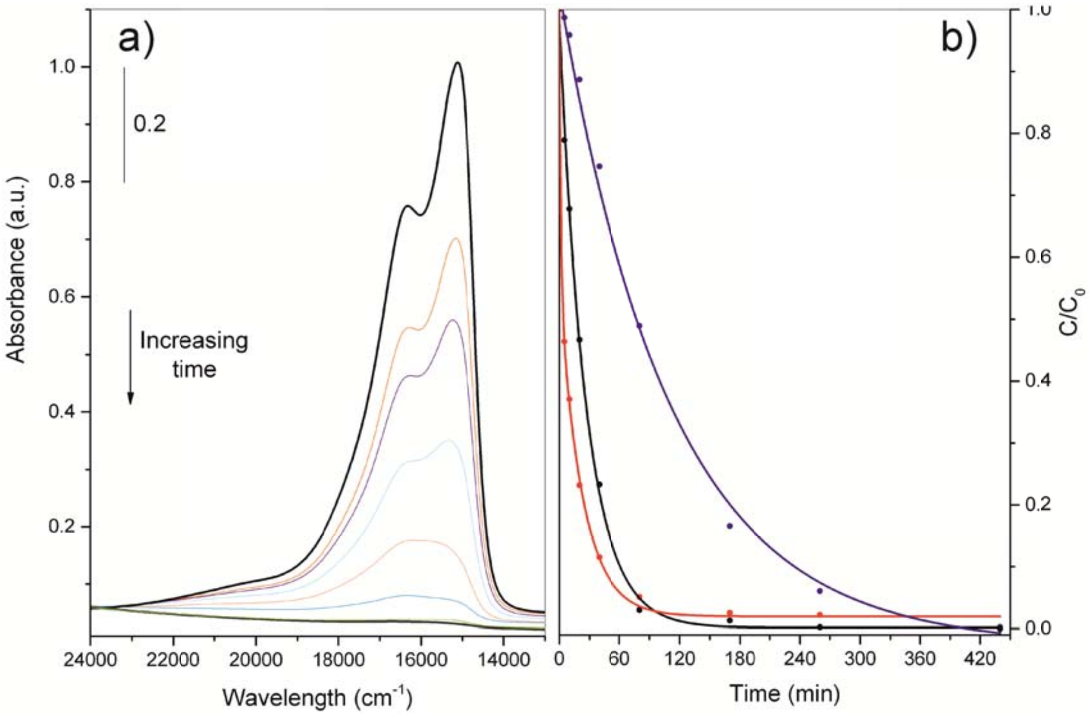

Figure S2. a) Evolution of UV-vis spectra of MB (water solution) adsorbed on MoS_2/TiO_2 as a function of the exposure time under visible light irradiation (0, 5, 10, 20, 40, 80, 170 and 260 min); b) time dependence upon light exposure of MB adsorbed on TiO_2/H_2S (blue curve), on MoS_2/TiO_2 (red curve) and on TiO_2 used as reference material (black curve).
